# Temporal constraints on leaf-level trait plasticity for next-generation land surface models

**DOI:** 10.1093/aob/mcaf045

**Published:** 2025-03-24

**Authors:** A Odé, N G Smith, K T Rebel, H J de Boer

**Affiliations:** Environmental Sciences, Copernicus Institute of Sustainable Development, Utrecht University, Princetonlaan 8a, 3584 CB Utrecht, The Netherlands; Department of Biological Sciences, Texas Tech University, 2901 Main St, Lubbock, TX 79409, USA; Environmental Sciences, Copernicus Institute of Sustainable Development, Utrecht University, Princetonlaan 8a, 3584 CB Utrecht, The Netherlands; Environmental Sciences, Copernicus Institute of Sustainable Development, Utrecht University, Princetonlaan 8a, 3584 CB Utrecht, The Netherlands

**Keywords:** Vegetation modelling, eco-evolutionary optimality, ecophysiology, photosynthesis, leaf functional traits, phenotypic plasticity, timescales, photosynthetic capacity, stomatal conductance, leaf gas exchange, leaf hydraulics

## Abstract

**Background and Aims:**

Dynamic global vegetation models (DGVMs) are essential for quantifying the role of terrestrial ecosystems in the Earth’s climate system, but struggle with uncertainty and complexity. Eco-evolutionary optimality (EEO) theory provides a promising approach to improve DGVMs based on the premise that leaf carbon gain is optimized with resource costs. However, the timescales at which plant traits can adjust to environmental changes have not yet been systematically incorporated in EEO-based models. Our aims were to identify temporal constraints on key leaf photosynthetic and leaf functional traits, and develop a conceptual framework for incorporation of temporal leaf trait dynamics in EEO-based models.

**Methods:**

We reviewed the scientific literature on temporal responses of leaf traits associated with stomata and hydraulics, photosynthetic biochemistry, and morphology and lifespan. Subsequent response times were categorized from fast to slow considering *physiological*, *phenotypic* (acclimation) and *evolutionary* (adaptation) mechanisms. We constructed a conceptual framework including several key leaf traits identified from the literature review. We considered temporal separation of dynamics in the leaf interior to atmospheric CO_2_ concentration (*c*_i_:*c*_a_) from the optimal *c*_i_:*c*_a_ ratio [χ_(optimal)_] and dynamics in stomatal conductance within the constraint of the anatomical maximum stomatal conductance (*g*_smax_). A proof-of-concept was provided by modelling temporally separated responses in these trait combinations to CO_2_ and humidity.

**Key Results:**

We identified 17 leaf traits crucial for EEO-based modelling and determined their response mechanisms and timescales. Physiological and phenotypic response mechanisms were considered most relevant for modelling EEO-based trait dynamics, while evolutionary constraints limit response ranges. Our conceptual framework demonstrated an approach to separate near-instantaneous physiological responses in *c*_i_:*c*_a_ from week-scale phenotypic responses in χ_(optimal)_, and to separate minute-scale physiological responses in stomatal conductance from annual-scale phenotypic responses in *g*_smax_.

**Conclusions:**

We highlight an opportunity to constrain leaf trait dynamics in EEO-based models based on physiological, phenotypic and evolutionary response mechanisms.

## INTRODUCTION

Plants are key players in the global carbon and hydrological cycles, and understanding their development, growth and survival is essential for reliably modelling future global vegetation dynamics and biosphere–atmosphere feedbacks in a changing climate (Prentice and Cowling, 2013). Leaves are the essential plant organs where carbon and water exchange takes place. Leaves can adapt dynamically to their environment on different timescales and through different mechanisms to maximize fitness (Harrison *et al.*, 2021), for example through fast physiological responses in stomatal aperture to follow diurnal environmental variation (McElwain *et al*., 2016), or through very long-term evolutionary processes to develop C_4_ photosynthesis (Sage, 2004). Dynamic global vegetation models (DGVMs) are used to simulate these leaf-level dynamics. Leaf-level photosynthesis in DGVMs is usually calculated with the Farquhar *et al.* (1980) model and is central to the quantification of net primary production (Prentice and Cowling, 2013; Prentice *et al.*, 2015). However, a limitation of current generation DGVMs is that leaf traits and subsequent vegetation characteristics typically use plant functional type (PFT)-dependent values for plant traits (Smith and Dukes, 2013; Prentice *et al.*, 2015; Mengoli *et al.*, 2022). A constraint of PFTs is that these are defined using predefined values of plant traits, although these plant traits actually exhibit great variation, even within a single PFT, as well as in their level of responsiveness to environmental changes (Wright *et al.*, 2004, 2005). Using PFTs, or indeed any categorical classification such as species means, therefore omits variability in traits that individual plants show in response to environmental changes through plastic responses.

A promising new approach to better include leaf trait dynamics in land surface models is based on eco-evolutionary optimality (EEO) theory (Medlyn *et al.*, 2011; Prentice *et al.*, 2014; Schymanski *et al.*, 2015; Franklin *et al.*, 2020; Harrison *et al.*, 2021). EEO theory states that plants adjust to their environment, thereby eliminating uncompetitive plant strategies by natural selection. At the leaf level, EEO theory states that trait combinations are selected that provide a combination of maximum carbon assimilation gain with minimal summed resource use cost (Wright *et al.*, 2003; Prentice *et al.*, 2014; Harrison *et al.*, 2021). Following this premise, EEO theory can be used to predict relationships between leaf-level traits and the environment. In resource-limited environments, plants may not achieve the predicted optimal trait combinations. However, the key point is that the species that will thrive are those that are relatively most fit for their environment compared to neighbouring species. So, they do not need to achieve optimality, as long as they are the ‘most optimal’ in their habitat.

Quantitative EEO theory has been proven to be a simple but powerful method for predicting and simulating leaf trait combinations under varying environmental conditions (Harrison *et al.*, 2021). For example, EEO theory can be used to predict traits along elevational gradients (Xu *et al.*, 2021*a*), coordination of photosynthesis and hydraulics traits along elevational gradients (Xu *et al.*, 2021*b*), leaf morphological traits along environmental gradients (Wang *et al.*, 2022), global maximum rates of carboxylation (*V*_cmax_) in C_3_ plants (Smith *et al.*, 2019), light-use efficiency of gross primary production (Stocker *et al.*, 2020) and the optimal leaf internal to ambient CO_2_ ratio (*c*_i_:*c*_a_) [χ_(optimal)_] across different environments (Prentice *et al.*, 2014), for explaining *V*_cmax_ and maximum rates of photosynthetic electron transport (*J*_max_) acclimation responses to temperature and CO_2_ in controlled experiments (Smith and Keenan, 2020), and the worldwide leaf economics spectrum (WLES) (Wang *et al.*, 2023). These key leaf traits change in response to the environment across timescales ranging from seconds to millions of years through a multitude of mechanisms, such as physiological responses, acclimation or long-term adaptation. Although steps are being made to separate sub-daily responses in stomatal conductance from acclimation of photosynthetic capacity at the week to monthly timescales (Mengoli *et al.*, 2022), further theoretical substantiation is needed to quantify different timescales to which leaves and, subsequently, plants and plant communities are able to respond to environmental changes.

**Fig. 1.**
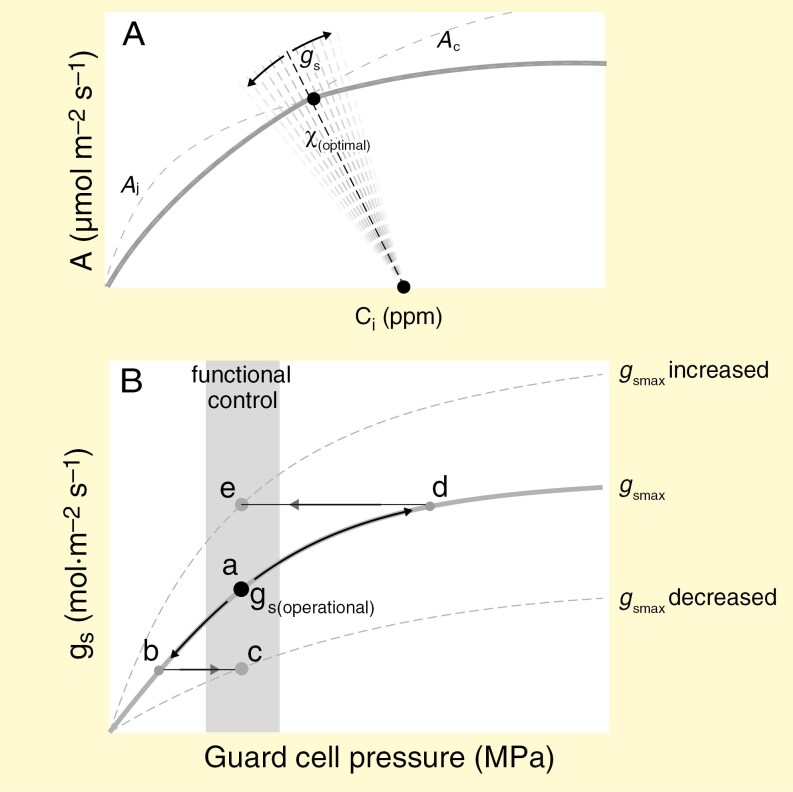
Existing frameworks of biochemical photosynthesis processes and physiological gas exchange. (A) General leaf photosynthesis–CO_2_ curve, illustrating the Rubisco-limited photosynthetic rate (*A*_c_) and the RuBP-limited photosynthetic rate (*A*_*j*_) as modelled with the Farquhar–von Caemmerer–Berry (FvCB) model, and the supply function of *g*_s(operational)_. *c*_i_ depends on *c*_a_, and χ_(optimal)_ acts as a setpoint for the dynamic, diurnal *g*_s_. (B) Physiological framework of stomatal adaptation adapted from Franks *et al.* (2012), with *g*_s(operational)_ corresponding to stomatal aperture, as a function of guard cell pressure. Stomata operate within the functional control region of the *g*_smax_ curve (diurnal *g*_s_), where points a, c and e correspond to γ, the conservative *g*_s_:*g*_smax_ ratio as described by McElwain *et al.* (2016). Environmental drivers can stimulate a decrease in *g*_s(operational)_ (a–b) on a timescale of weeks, driving *g*_s(operational)_ outside the functional control region. In order to return *g*_s(operational)_ to the functional control zone while conserving γ, leaves adjust their *g*_smax_ on a developmental timescale with the emergence of new leaves (b–c). In turn, when environmental stimuli drive an increase in *g*_s(operational)_ (a–d) to outside the functional control zone, leaves will increase their *g*_smax_ to conserve γ (d–e).

Key leaf traits that are generally used by EEO models include traits of photosynthetic biochemistry, namely *V*_cmax_ and *J*_max_, and stomatal conductance (*g*_s_). *V*_cmax_, *J*_max_ and *g*_s_, in combination with environmental conditions including vapour pressure deficit (VPD), light, temperature, atmospheric CO_2_ (*c*_a_) and atmospheric pressure, determine the net photosynthesis rate in relation to the leaf interior CO_2_ concentration (*c*_i_) (Fig. 1A). In the EEO theory-based ‘P-model’ (Prentice *et al.*, 2014) a key trait is χ_(optimal)_, which is defined as the optimal *c*_i_:*c*_a_ ratio, and is a time-averaged value of *c*_i_:*c*_a_ due to subdaily fluctuations of *g*_s_ (Fig. 1A). In addition, *g*_s_ is positioned on the transition between *V*_cmax_ and *J*_max_ as described by the coordination hypothesis (Chen *et al.*, 1993; Maire *et al.*, 2012; Quebbeman and Ramirez, 2016; Wang *et al.*, 2017). According to the P-model, χ_(optimal)_ is determined by *V*_cmax_, and a *g*_s_ that in the P-model is acclimated to *V*_cmax_ and therefore also acts on the same timescale as *V*_cmax_ (a week to a month) (Stocker *et al.*, 2020) (Fig. 1A).

In addition, a well-established theoretical framework for leaf gas exchange traits describes the relationship between the diurnally fluctuating *g*_s_, the operational stomatal conductance [*g*_s(operational)_] and the anatomical maximum conductance (*g*_smax_) (Franks *et al.*, 2012; McElwain *et al.*, 2016). According to this theory, *g*_s(operational)_ acts as a setpoint where the diurnal *g*_s_ fluctuates dynamically on a subdaily timescale (Fig. 1A). The parameter *g*_s(operational)_ is termed *g*_c(op)_ in Franks *et al.* (2012) and *g*_op_ in McElwain *et al.* (2016). The term *g*_smax_ is defined by the leaf stomatal morphology and density, where larger, fewer stomata result in a lower *g*_smax_ than smaller, more numerous stomata (Franks *et al.*, 2012; de Boer *et al.*, 2016). In this framework, the relationship between *g*_s(operational)_ and *g*_smax_ is presented by a saturating response function to guard cell pressure (Fig. 1B). McElwain *et al.* (2016) and Murray *et al.* (2020) found that plants typically operate at ~25 % of their *g*_smax_ to maximize the sensitivity of *g*_s_ to guard cell pressure (Fig. 1B, ‘region of functional control’), resulting in a conservative ratio of *g*_s(operational)_:*g*_smax_ of ~0.25 across species, which we will term γ throughout this paper (Fig. 1B).

Moreover, leaf photosynthetic and stomatal traits are fundamentally linked to leaf hydraulics and leaf morphology. Brodribb *et al.* (2007) found a consistent positive relationship between evolutionary increases in leaf hydraulic conductance (*K*_leaf_) and light-saturated photosynthesis (*A*_sat_). Here, higher *K*_leaf_ is required to sustain higher rates of transpiration associated with higher *g*_s_ and *g*_smax_, and associated with evolutionary developments in photosynthetic capacity (Flexas and Carriquí, 2020). Wright *et al.* (2004) describe the WLES, which has become a widely accepted framework for classifying leaf resource investment strategies on a spectrum from quick turnover rates with high productivity (fast) to slow turnover rates with lower productivity (slow). The WLES is accompanied by leaf-level traits of photosynthetic capacities, hydraulic capacities, leaf morphology and leaf lifespan, which implies underlying coordination following EEO principles (Wang *et al.*, 2023).

Clearly, leaf gas exchange and underlying investments in biochemistry and water transport are determined by leaf traits associated with stomata and hydraulics, biochemistry and morphology. However, the timescales at which these traits adjust to environmental changes are not yet systematically incorporated in EEO-based models. Our aims were to identify temporal constraints on key leaf photosynthetic and leaf functional traits, and develop a conceptual framework for incorporation of distinct timescales of leaf trait dynamics in EEO-based models. For this, we first reviewed the scientific literature on the timescales of leaf trait responses associated with their stomata and hydraulics, photosynthetic biochemistry, and leaf morphology and leaf lifespan. Subsequent response times were categorized from fast to slow considering physiological, phenotypic (acclimation) and evolutionary (adaptation) mechanisms. Second, we used the EEO-based P-model and principles from stomatal anatomy to develop a conceptual framework to separate temporal dynamics in leaf gas exchange associated with biochemistry and transpiration. Third, we used this framework to provide a proof-of-concept to model temporally separated responses in biochemistry and transpiration to changes in ambient CO_2_ and humidity. This framework can be used to develop improved EEO schemes for DGVMs to better capture the temporal dynamics of leaf gas exchange and underlying investments in biochemistry and water transport.

## METHODOLOGY

### Literature review and categorization of response timescales

**Fig. 2.**
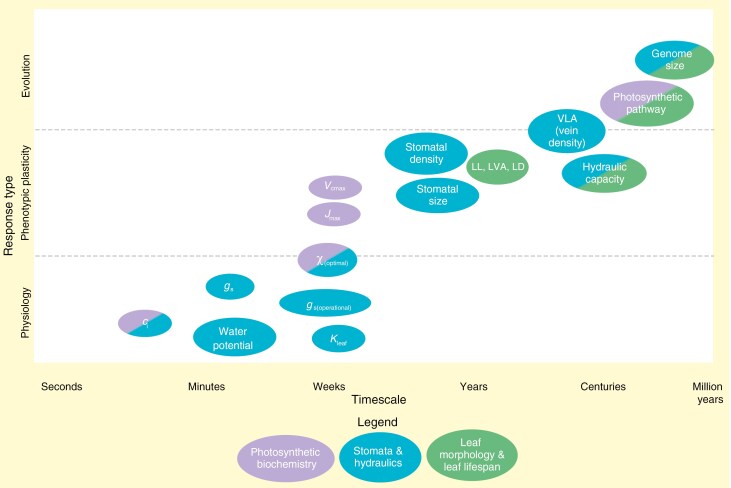
Key leaf traits and their corresponding timescale and response type as derived from the literature review. Colours correspond to the structure as used in the literature review, where two colours mean that a trait can be categorized in both.

Key leaf traits relevant for EEO modelling were identified from published literature, with their corresponding description and units. Literature search queries were composed of the selected traits plus characteristics of adjustment, for example ‘stomatal conductance acclimation’, ‘stomatal conductance adaptation’ and ‘*g*_smax_ evolution’. Current knowledge on responses of these traits on different timescales was reviewed and structured by categorizing each trait as ‘stomata and hydraulics’, ‘photosynthetic biochemistry’ or ‘leaf morphology and leaf lifespan’. Within each of these categories, trait responses were structured by (1) responses in physiological processes, (2) responses through developmental processes that result from phenotypic plasticity (acclimation) and (3) evolution (adaptation) (Fig. 2).

### Conceptual framework to separate timescales in trait responses

Building from our literature results, we developed a conceptual framework to quantify and separate temporal dynamics of idealized leaf trait responses. On the *y*-axis we plot the ratio of *c*_i_:*c*_a_ to χ_(optimal)_, and on the *x*-axis we plot the ratio *g*_s_:*g*_smax_. This enables us to separate faster responses of *c*_i_ and *g*_s_ from slower responses of χ_(optimal)_ and *g*_smax_. To simulate leaf trait responses, we combine three well-established models: a photosynthesis model based on Farquhar *et al.* (1980), a model for instantaneous *g*_s_ responses from Medlyn *et al.* (2011), and the EEO-based ‘P-model’ for acclimation in leaf biochemistry and morphology (Stocker *et al.*, 2020). Note that the predicted optimal *g*_s_ from Medlyn *et al.* (2011) describes the diurnally fluctuating *g*_s_, and that the EEO-based P-model of Stocker *et al.* (2020) predicts an optimal *g*_s_ acclimated to *V*_cmax_.

### Framework proof-of-concept and simulations

As a proof-of-concept for our theoretical framework, we simulated the trait responses across timescales to instantaneous changes in two theoretical scenarios of environmental change: CO_2_ increase and VPD increase. The scenarios represent an instantaneous increase in CO_2_ from 400 to 800 ppm, and in VPD from 1 to 2 kPa, while keeping all other environmental variables constant. Initial values were based on typical unstressed ambient growth conditions under current temperate climate [temperature 25 °C (298.15 K), light 800 µmol m^−2^ s^−1^]. Increases considered a doubling of initial values for CO_2_ and VPD. A detailed approach to the scenario modelling and the complete code is provided in the Supplementary Data.

**Fig. 3.**
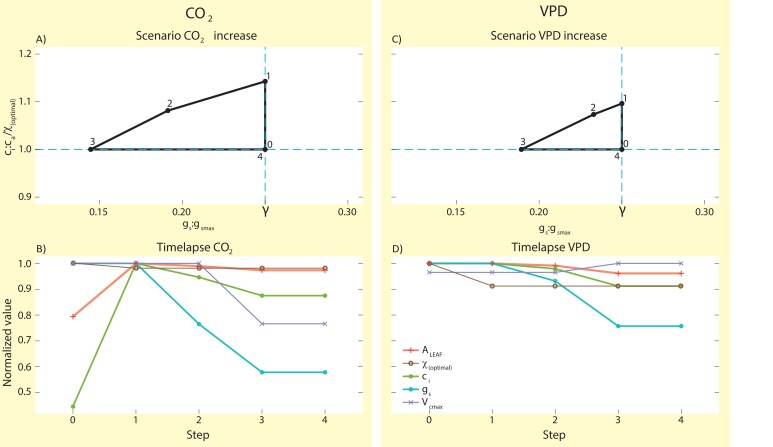
Modelled scenarios to the new conceptual framework of idealized leaf gas exchange across timescales. The *x*-axis is the *g*_s_:*g*_smax_ ratio and the assumed optimal γ, and the *y*-axis describes *c*_i_:*c*_a_/χ_(optimal)_. The time-lapses illustrate individual leaf traits normalized to the maximum value on a scale of 0 to 1 over the time steps of the scenarios. Step 0 represents the initial optimized trait combination. Step 0–1 represents the near-instantaneous (seconds) response, 1–2 represents the physiological response in stomatal aperture (minutes), 2–3 represents biochemical acclimation of photosynthetic capacity (weeks) and 3–4 represents leaf developmental adjustment of stomatal anatomy (seasonal and longer). Step 4 represents the new optimized trait combination. (A) Scenario of *c*_a_ increase from 400 to 800 ppm. (B) Time-lapse of individual leaf traits *A*_leaf_, χ_(optimal)_  *c*_i_, *g*_s_ and *V*_cmax_ for the CO_2_ increase scenario. (C) Scenario of VPD increase from 1 to 2 kPa. (D) Time-lapse of individual leaf traits *A*_leaf_, χ_(optimal),_  *c*_i_, *g*_s_ and *V*_cmax_ for the VPD increase scenario.

To illustrate the separate leaf trait changes through time for the framework scenarios, we constructed a time-lapse figure for each scenario (Fig. 3B, D). The time-lapse figures display how the values of the photosynthetic rate per leaf area (*A*_leaf_), χ_(optimal),_  *c*_i_, *g*_s_ and *V*_cmax_ change stepwise on different timescales in the corresponding scenarios. For each trait within each scenario, the values were normalized to the maximum value, to create normalized values on a scale from 0 to 1. ChatGPT, powered by GPT-4, was used as a tool for a few model code lines to optimize the correct data format, in order to plot the time-lapse graphs (lines 115–130, 222–237). Optimized code output was checked against manual calculations to ensure correct outcomes.

## RESULTS

### Timescales of leaf trait dynamics

#### Stomata and leaf hydraulics.

The exchange of water and carbon dioxide at the leaf surface is fundamentally controlled by gas exchange across the stomata and the resulting *g*_s_. Physiologically driven changes in *g*_s_ can be controlled by stomatal aperture, which is regulated by changes in guard cell turgor through ion influx and/or leaf moisture levels (McAdam and Brodribb, 2014). Also, *g*_s_, together with CO_2_ drawdown, plays an essential role in regulating *c*_i_. Research over the past few decades has shown that there are two types of stomatal regulation: passive, mediated by hydraulic closure in response to leaf water status, and active, mediated by changing levels of the phytohormone abscisic acid which triggers osmotic ion efflux to depolarize the guard cell membranes (McAdam and Brodribb, 2014; Clark *et al.*, 2022). External stimuli for opening and closing the stomata are light quality and quantity, atmospheric CO_2_ concentration, VPD and soil moisture status. Research has shown that the rate of stomatal opening and closing is variable between species and species groups, and depends on stomatal morphology, stomatal size, plant functional type and climate (Vico *et al.*, 2011; Drake *et al.*, 2013; Elliott-Kingston *et al.*, 2016). For example, Vico *et al.* (2011) found stomatal responses to changing light environment ranging from 5 to 30 min across a variety of species. They also found that species from dry climates as well as grasses with dumbbell-shaped stomata have faster response rates. Additionally, Elliott-Kingston *et al.* (2016) found mean half-closure times of stomata from light to dark of six species ranging from 7 to 30 min, and one species (*Ginkgo biloba*) shown to be the slowest with a half-closure time of 1 h 45 min. They also found that species that diversified under low CO_2_ concentrations close their stomata faster compared to those diversified in a high CO_2_ world, indicating selection pressure for stomatal closing rates during taxon diversification to optimize water use efficiency. Drake *et al.* (2013) found that the maximum rate of stomatal opening and closing as well as *g*_s(operational)_ are negatively linked to stomatal size across species of *Banksia*. This shows a mechanistic link between stomatal opening/closing rates and stomatal morphology. Across plant taxa, Elliott-Kingston *et al.* (2016) found no correlation between stomatal size and closing in response to darkness. Although physiological response rates of *g*_s_ can thus be influenced by the environment during diversification and/or stomatal morphology, we conclude that the timescale of *g*_s_ responses falls consistently within minutes to hours (Fig. 2). We do not consider mesophyll conductance in detail in this study, since it is currently not incorporated in the P-model. However, future model development should consider mesophyll conductance since it can have large effects on photosynthetic nitrogen use efficiency and water use efficiency, and respond on different timescales (Buckley and Warren, 2014). Levels of *c*_i_ respond nearly instantaneously to changes in *c*_a_, and are jointly controlled by stomatal conductance and the rate of CO_2_ drawdown, so we therefore also place *c*_i_ on a timescale of seconds to hours.

Stomata can remain open if the leaf water status is sufficient to prevent desiccation and leaf embolism, which depends on the leaf water potential (Ψ_leaf_) and hydraulic conductance (*K*_leaf_) Sack and Holbrook, (2006). Stomatal regulation of Ψ_leaf_ includes enhancing the rate of transpiration, causing a more negative leaf water potential, leading to passive water uptake by the roots in the soil. Due to the coupling with stomata, Ψ_leaf_ and *K*_leaf_ show a similar diurnal pattern as stomatal conductance (Lo Gullo *et al.*, 2005; Sack and Holbrook, 2006). When a leaf dehydrates, *K*_leaf_ and Ψ_leaf_ will decline (become more negative), triggering the closing of stomata, resulting in a decrease of *g*_s_ and transpiration and an increase (less negative) in Ψ_leaf_. *K*_leaf_ will therefore be linked to the fast, diurnal dynamics of leaf water status and *g*_s_. Gradual dehydration over an extended period will lead to a long-term decrease in *K*_leaf_. Owing to the different possible rates of dehydration, *K*_leaf_ was found to be dynamic over a timescale from minutes to months (Sack and Holbrook, 2006). The stringency of stomatal control on Ψ_leaf_ can be described as falling along an isohydric–anisohydric continuum (Salvi *et al.*, 2022), whereby towards the isohydric end plants exhibit tight control over stomatal closing during drought (‘conservative behaviour’), which stabilizes Ψ_leaf_ (less variation). Towards the other end, anisohydric species maintain stomatal conductance during drought to sustain photosynthetic rates, resulting in greater Ψ_leaf_ variations with changes in soil water availability (‘risk-taking behaviour’). The level of isohydry and stomatal behaviour is influenced by hydraulic traits of the xylem (Klein, 2014), and environmental conditions, including competition for water and soil processes (Lu *et al.*, 2016; Mrad *et al.*, 2019). We conclude that Ψ_leaf_ acts on a timescale similar to the physiological responses of the stomata, ranging from minutes to hours, whereas *K*_leaf_ acts on a timescale of minutes to months (Fig. 2).

Although *g*_s_ can change at the timescale of minutes to hours, its responses are constrained at the upper limit by *g*_smax_ (Franks and Beerling, 2009; Franks *et al.*, 2012). Plants have the ability to modify *g*_smax_ by adjusting stomatal density, pore length and pore depth with the emergence of every new leaf. This phenotypic plasticity enables them to adjust to distinct environmental drivers, such as climate gradients within canopies (Boardman, 1977; Brodribb and Jordan, 2011; Campany *et al.*, 2016; Dörken and Lepetit, 2018). Stomatal density appears to be coordinated with vein density when acclimating to variable canopy light availability, which is at least in some species regulated by leaf size to preserve leaf hydraulic conductance and stomatal conductance proportionality (Brodribb and Jordan, 2011; Carins Murphy *et al.*, 2012, 2014). High vein length per unit area (VLA, ‘vein density’) varies strongly across species, and can enable higher *K*_leaf_ and *g*_s_ per unit leaf area (Sack and Scoffoni, 2013). Also, Drake *et al.* (2019) found that VLA is less plastic than stomatal density per unit area within lineages. The plastic responses of stomata and hydraulics are, however, not consistently clear and may take multiple seasons to be fully revealed, so phenotypic plasticity responses may overlap with evolutionary responses in genotypes (Hincke *et al.*, 2016). So, we conclude that the response of *g*_smax_ occurs on a timescale of years to decades, whereas VLA acts on a longer timescale of years to centuries.

There is a negative relationship between stomatal size and density across species and evolutionary groups (Franks and Beerling, 2009; Franks *et al.*, 2009; De Boer *et al.*, 2011; Lammertsma *et al.*, 2011), which creates a range of possible *g*_smax_ values (Zhang *et al.*, 2021), whereby the highest values of *g*_smax_ can only be attained with a combination of high stomatal density and small stomatal sizes. This relationship reflects a combination of geometric constraints (changes in density caused by size) and non-geometric constraints (trade-off between space allocation to stomata on the leaf surface and increasing *g*_smax_) (de Boer *et al.*, 2016; Zhang *et al.*, 2021). Environmental drivers can impose selection pressures on *g*_smax_ within plant communities, resulting in adaptations and speciation of, for example, dry-adapted species with low *g*_smax_ values (Taylor *et al.*, 2012). On a macro-evolutionary timescale, older lineages such as gymnosperms and ferns appear to have larger and fewer stomata due to historical high CO_2_ levels at their time of emergence, resulting in lower *g*_smax_ values, while the younger lineage of angiosperms evolved smaller, denser stomata to cope with ‘CO_2_ starvation’ to retain higher *g*_smax_ values (De Boer *et al.*, 2011; Lammertsma *et al.*, 2011). So, *g*_smax_ is constrained by stomatal (guard cell) size.

The hydraulic capacity of a leaf, i.e. its maximum leaf hydraulic conductance, is determined by anatomical properties and links directly to mesophyll conductance, vein architecture and xylem conductance (Sack and Holbrook, 2006). There is high variability in hydraulic capacity across species (65-fold) and within life forms (10-fold), ranging from the lowest in conifers and pteridophytes to the highest in angiosperms and agricultural plants (Sack and Holbrook, 2006). The coordination of increases in *g*_smax_ and hydraulic features allows plants to make substantial changes to these traits while preserving functional links (Brodribb *et al.*, 2007, 2013; Mcelwain *et al.*, 2016). Large increases in these traits proved to be an evolutionary advantage, as shown for the angiosperm revolution where vein density increase enabled an increase in *g*_smax_, thereby enabling higher gas exchange capacities (Feild *et al.*, 2011; De Boer *et al.*, 2012). The maximum light-saturated photosynthetic capacity (*A*_sat_) is linked to the hydraulic conductance of leaves so that, on an evolutionary timescale, the development of increased *K*_leaf_ by changes in vein properties and vein positioning is linked to an increase in *A*_sat_ (Brodribb *et al.*, 2005, 2007, 2017; Scoffoni *et al.*, 2016). This coordination between photosynthesis and hydraulic traits is observed across diverse lineages (Brodribb *et al.*, 2005), as well as within lineages (Scoffoni *et al.*, 2016). A potential driver for unified changes in stomatal and hydraulic leaf traits is cell size, which in turn could be mediated by changes in genome size (Brodribb *et al.*, 2013; Roddy *et al.*, 2020). We conclude that hydraulic capacity adjusts on a timescale of decades to centuries.

#### Photosynthetic biochemistry

Biochemical photosynthesis reactions of C_3_ plants take place within the chlorophyll-containing chloroplasts (Farquhar *et al.*, 1980). These biochemical reactions are limited by either ribulose-1,5-bisphosphate (RuBP) carboxylation or RuBP regeneration, or triose-phosphate utilization (TPU), which are influenced by their respective maximum rate parameters, *V*_cmax_ and *J*_max_ and TPU rate. TPU limitation occurs only at very high CO_2_ levels, high light levels and/or low temperatures and is therefore generally assumed negligible in photosynthesis models (Lombardozzi *et al.*, 2018). Rubisco, the key enzyme catalysing the carboxylation reaction, contains a large amount of nitrogen and is therefore found to scale with leaf nitrogen per area (*N*_area_). This relationship between *V*_cmax_ and leaf nitrogen is commonly used to estimate *V*_cmax_ from *N*_area_ (Wright *et al.*, 2004; Kattge *et al.*, 2009; Walker *et al.*, 2014). However, Luo *et al.* (2021) argue that using linear relationships between *N*_area_ and *V*_cmax_ neglects the variation in the fraction of leaf nitrogen allocated to Rubisco, which is a highly plastic trait (Waring *et al.*, 2023) that causes high variability in model estimates of *V*_cmax_. Photosynthesis is stimulated by increased atmospheric CO_2_ by a fast response of the release of the CO_2_ diffusion limitation (‘CO_2_ fertilization’). CO_2_ fertilization therefore leads to a short-term increase in photosynthesis rates (Smith and Dukes, 2013). Enhanced light quantity will increase the photosynthesis rate due to the increased production of ATP from light-dependent reactions needed to support Calvin cycle processes (Leverenz *et al.*, 1990). When the light-dependent reactions are saturated, excess energy will be dissipated into heat by a molecular adaptation process of non-photochemical chlorophyll fluorescence quenching (NPQ), to prevent photodamage to the reaction centre of photosystem II (PSII) and potentially antenna pigments (Kono and Terashima, 2014; Ruban, 2016).

Plants show the ability to alter their photosynthetic traits in response to temperature, so that their (new) optimum temperature for photosynthesis corresponds to the growth temperature to enhance photosynthetic rates (Sage and Kubien, 2007). Growth temperature alters the enzymatic temperature dependence of the biochemical photosynthetic rate components *V*_cmax_ and *J*_max_, where acclimation to warming results in a positive shift of the optimum temperature of *V*_cmax_ and *J*_max_ (Hikosaka *et al.*, 2006; Way and Yamori, 2014; Smith and Keenan, 2020). Plants show, in general, levels of phenotypic plasticity in this acclimation process, which may be linked to increased enzyme heat tolerance, metabolic enzyme allocation, changes in the activation energy of *V*_cmax_ and *J*_max_, the ratio of *J*_max_ to *V*_cmax_ (decreases at higher growth temperatures), and/or stomatal adjustment (Sage and Kubien, 2007; Yamori *et al.*, 2014; Smith and Keenan, 2020). Shifts in temperature optima may be coordinated by leaf nitrogen partitioning, where leaf nitrogen use efficiency is hypothesized to be at maximum when the photosynthetic rate is co-limited at the growth (optimum) temperature. CO_2_ acclimation, after the initial short-term CO_2_ fertilization, consists of downregulation of *V*_cmax_. This response occurs as plants decrease the density of CO_2_-fixing enzymes, due to the increased substrate for Rubisco, and also reduce stomatal conductance to minimize water loss (Bazzaz, 1990; Smith and Keenan, 2020). Also, resource use for *V*_cmax_ and *J*_max_ are reduced under elevated CO_2_, with a reduction in *J*_max_ relative to *V*_cmax_ (Smith and Keenan, 2020). Light quantity also influences investment in the photosynthetic apparatus, where in low light, leaves invest in light capture organelles (chlorophyll antenna) and less in photosynthetic enzymes. Plants grown under high light levels, in contrast, invest more in photosynthetic enzymes, resulting in higher photosynthetic capacity than plants acclimated to low light (Boardman, 1977). The timescale of photosynthetic acclimation ranges from days (e.g. optimum temperature shifts; Smith and Dukes, 2017) to weeks or longer (Smith and Dukes, 2013), due to the nature of change as well as the environmental change to which the plant acclimates (Smith, 2024). We therefore place the acclimation of *V*_cmax_ and *J*_max_ on a timescale of weeks.

On an evolutionary timescale, selection pressures can drive changes in photosynthetic mechanisms to optimize to their environment. C_4_ photosynthesis is a collective term for a series of biochemical and anatomical adjustments of the leaf to concentrate phosphoenolpyruvate carboxylase (PEPC) and around Rubisco, as an addition to C_3_ photosynthesis (Christin and Osborne, 2014). It is facilitated by the characteristic Kranz anatomy, which separates the steps of carbon fixation and the Rubisco reaction (Sage, 2004; ). It is thought that C_4_ photosynthesis evolved as a response to declining atmospheric CO_2_ concentrations since the Carboniferous to deal with increased photorespiration (Sage, 2004). Although there are many versions of C_4_ photosynthesis (enzymatic and anatomical variations), there is an overlapping enzymatic step in all of them: the carboxylation reaction to yield oxaloacetic acid (OAA), catalysed by PEPC. This carboxylation step takes place in an outer layer of cells derived from mesophyll tissue. The produced four-carbon acids then move to the location of Rubisco, the bundle sheath cells, where CO_2_ is released from decarboxylation. This results in increased concentrations of CO_2_ levels by 10- to 100-fold compared to ambient air in the compartment which then nearly saturates Rubisco (Yamori *et al.*, 2014). Due to this concentrating mechanism of CO_2_ around Rubisco, C_4_ photosynthesis can overcome high rates of photorespiration in challenging environments, such as high VPD, saline and low nutrient conditions. It is thought that this enables C_4_ species to establish is such environments due to their enhanced nitrogen- and water use efficiency compared to C_3_ species (Sage, 2004).

Another auxiliary mechanism to C_3_ photosynthesis is crassulacean acid metabolism (CAM photosynthesis), which generally occurs as an adaptation to (semi)arid environments (Silvera *et al.*, 2010). Where C_4_ photosynthesis separates carbon fixation and the Rubisco reaction in space, CAM photosynthesis separates this in time between day and night. CAM plants fix CO_2_ during the night when temperatures are lower and water loss is minimized. Carbon is fixed by PEPC, and the products (malic acid and citric acid) are stored in the vacuole for decarboxylation and sugar production during the day, facilitated by Rubisco. In this way, the stomata can remain relatively closed during the day to prevent excessive water loss (Silvera *et al.*, 2010). CAM photosynthesis also evolved multiple times independently from C_3_ plant ancestors (Bräutigam *et al.*, 2017; Sage *et al.*, 2023). C_4_ and CAM photosynthetic pathways require extensive anatomical and biochemical changes over an evolutionary timescale, and therefore the trait of ‘photosynthetic pathway’ is placed on a timescale of centuries to millions of years.

#### Leaf morphology and leaf lifespan

Leaf morphology is typically determined at the start of leaf growth. Since this is a structural trait, responses mainly happen at longer developmental and evolutionary timescales. Intraspecific plasticity of leaf dry mass per area (LMA), in combination with leaf lifespan (LL), enables leaves to respond to environmental changes to maintain positive net carbon gains (Onoda *et al.*, 2017). Leaves with higher LMA require either high photosynthetic rates or high LL to return these construction costs. This optimization strategy is in line with EEO theory (Wang *et al.*, 2023). LMA can be broken down into leaf density (LD) and leaf volume per area (LVA) so that LMA = LVA × LD (Poorter *et al.*, 2009). The main drivers of phenotypic responses in LD, LVA and LL are light quantity (primary), CO_2_ levels, temperature and (to a lesser extent) soil nutrients (Poorter *et al.*, 2009). In general, in low-light conditions, leaf area increases to capture more light, and also to enable the leaf to capture all the light with fewer cell layers (Gratani *et al.*, 2006). In high-light conditions, a leaf increases in thickness to contain more photosynthetic biomass that the light can reach, and this also increases photosynthetic capacity. At least in tropical tree species, this is also accompanied by increased vascular cells to meet higher transpirational water demands (Russo and Kitajima, 2016). Light-driven plasticity was also found for leaf nitrogen content per area, and photosynthetic capacity per leaf area (Keenan and Niinemets, 2016). CO_2_ drives an increase in LMA, induced by increases in both LD and LVA (Poorter *et al.*, 2009). This is mainly an effect of starch accumulation, and when excluding total non-structural carbohydrates (TNCs), effects were found to be small or non-existent. LMA also increases in response to low temperatures, resulting from a limitation on cell expansion leading to higher LD (Atkin *et al.*, 2006; Poorter *et al.*, 2009). In return, LMA decreases at higher temperatures, although not linearly. Nutrient limitation has a moderate effect on LMA, with the direction of the response, either positive or negative, depending on various factors, and only becomes notable at severe limitation levels (Poorter *et al.*, 2009).

High phenotypic plasticity can be observed within plant canopies, since leaf traits are highly sensitive to light conditions. Canopies are also characterized by gradients in CO_2_ concentration, humidity and temperature (Niinemets *et al.*, 2015). Shaded leaves lower in the canopy are generally larger and thinner and have a lower fresh and dry weight per leaf area than sun leaves (Boardman, 1977). Sun leaves have a higher LVA due to an increase in the number of cell layers in mesophyll tissue, cell elongation in the palisade tissue or a combination thereof (Hoshino *et al.*, 2019), which also increase photosynthetic capacity due to increased chloroplasts (Evans and Poorter, 2001). Mass-based assimilation rate, respiration rate and LMA are therefore generally found to be lower for shade leaves compared to sun leaves (Chen *et al.*, 2020). Plasticity in LL has not yet been researched extensively, but Vincent (2006), for example, found a similar magnitude of plasticity in LL as in LMA in tropical seedlings, where leaves grown in low light have a longer lifespan. This was explained by slower ageing of shaded leaves, which also have slower metabolic rates (Vincent, 2006). Although phenotypic plasticity in LMA and LL is thus generally found, Dong *et al.* (2020) found that LMA and LL, and especially leaf area, appear to have much less plasticity than stomatal, hydraulic and biochemical traits along environmental gradients, probably due to their structural nature (Harrison *et al.*, 2021) or possibly through genetics related to cell size on an evolutionary timescale (Brodribb *et al.*, 2013) and these traits are thus less plastic on shorter acclimation timescales. In conclusion, though morphological traits are difficult to assign a specific timescale to, they typically occur on developmental and longer timescales, and we therefore place LVA, LD and LL on a timescale of years to centuries. Since morphological traits are linked to cell size, which is in turn linked to genome size and acts on a timescale of millions of years (Faizullah *et al.*, 2021), we add genome size as a trait in our overview on an evolutionary timescale (Fig. 2).

On an evolutionary timescale, an interspecific conservative range of leaf traits is described by the WLES. The WLES is a concept that describes a conservative range of leaf trait combinations that plants exhibit over different environments and habitats on a population level (Wright *et al.*, 2004), and has recently been explained by EEO principles (Wang *et al.*, 2023). The WLES consists of six key leaf traits: LMA, LL, photosynthetic assimilation rate per dry mass (*A*_mass_), dark respiration rate per dry mass (*R*_mass_), leaf nitrogen concentration per dry mass (*N*_mass_) and leaf phosphorus concentration per dry mass (*P*_mass_). On one end of the spectrum are leaves with quick turnover rates, with high leaf nutrient concentrations, high photosynthetic rates, high respiration rates, short LL and low LMA. On the other end of the WLES are the slow-return leaves with long LL, high LMA, low nutrient concentrations, and low *A*_mass_ and *R*_mass_ (Wright *et al.*, 2004). The negative correlation between LMA and *A*_mass_ is explained by the longer diffusion pathway between stomata and chloroplasts, which decreases mesophyll conductance, and shadowing of underlying chloroplasts. Research by Onoda *et al.* (2017) showed negative correlations of LMA with *N*_area_ and within-leaf CO_2_ diffusion rates, caused by thicker mesophyll cell walls, which supports this hypothesis.

The link between LMA and LL reflects leaf economic strategies, with LMA reflecting the costs and LL reflecting the timespan of carbon gain. Note that on a phenotypic plasticity level, acclimation within a plant to sun–shade results in high LMA and low LL for sun-acclimated leaves, due to more cell layers to utilize the high amount of incoming light and fast carbon overturn rates, compared to shade-acclimated leaves with low LMA (relatively large leaf area to capture more light) (Gratani *et al.*, 2006). In contrast, on an evolutionary WLES timescale, adaptation of LMA results from resource-limited environments: leaves of shade-tolerant species have higher LMA with higher LL than light-demanding species. This is explained by leaf economics: in an environment where resource costs are high, it takes a longer time to return leaf construction costs, hence the higher LL (Russo and Kitajima, 2016). EEO theory appears suitable to provide an explanation for the WLES of woody plant species from environmental variables, specifically for opposing latitudinal trends in LMA between deciduous and evergreen leaves. This is explained and tested by leaf-level maximization of life cycle average net carbon gain (Wang *et al.*, 2023). So, the WLES is not a distinct leaf trait, but rather explains the interspecific conservative relationship between leaf traits on an evolutionary timescale in a leaf economics context, which is in line with EEO theory, and therefore integrated in our conceptual framework.

### Identified key leaf traits and corresponding timescale of response

Our literature review identified the key traits for EEO-based leaf-level models, quantified the timescales of their environmental responses, ranging from seconds to millions of years, and categorized their corresponding response mechanisms as physiological, phenotypic (acclimation) or evolutionary (adaptation). An overview of identified key leaf traits with their definition, units and key citations can be found in Supplementary Data Table S1. These traits and their responses are visualized in Fig. 2.

### Idealized responses in leaf gas exchange across timescale

We simulated responses of the key leaf traits we identified in the literature review to instantaneous increases in CO_2_ and VPD as a proof-of-concept. We separate the timescale of responsiveness between traits in relatively fast physiological responses (*c*_i_ and *g*_s_), and slower acclimation responses within phenotypic plasticity (*V*_cmax_ and *g*_smax_). We hypothesize from our review that an increase in CO_2_ will lead to a net increase in *A*_leaf_ while decreasing *V*_cmax_ and *g*_s_, and an increase in VPD will decrease *A*_leaf_ through a decrease in *g*_s_ to prevent leaf desiccation.

Exploring the modelled responses of (normalized) trait values across separate timescales revealed that an increase in CO_2_ from the initial 400 to 800 ppm (step 0–1 in Fig. 3A) resulted in an instantaneous increase in *c*_i_:*c*_a_ due to the instantaneous increase in *c*_i_. As χ_(optimal)_ decreased by 1.9 % under elevated CO_2_ (brown line in Fig. 3B) and *c*_i_ increased by 124 % (green line in Fig. 3B), the instantaneous response to increased CO_2_ increased the ratio *c*_i_:*c*_a_/χ_(optimal)_ from the initial optimal by 14 % from 1 to 1.14 (step 0–1 in Fig. 3A). The subsequent physiological response in stomatal aperture occurring at a timescale of minutes (step 1–2 in Fig. 3A, blue line in Fig. 3B) led to a decrease in the ratio *c*_i_:*c*_a_/χ_(optimal)_ by 5.4 % (green line in Fig. 3B), and a decrease in stomatal conductance by 23.5 % (blue line in Fig. 3B). Consequently, the ratio *g*_s_:*g*_smax_ was moved below the long-term optimal γ (blue dashed line in Fig. 3A). Acclimation of leaf photosynthetic capacity (step 2–3 in Fig. 3A) decreased *V*_cmax_ by 23.4 % (purple line in Fig. 3B), accompanied by a decrease in *c*_i_:*c*_a_/χ_(optimal)_ of 7.5 % (green line in Fig. 3B), and a decrease in *g*_s_ of 24.4 % (blue line in Fig. 3B). Our combination of models also predicted a coordination of downregulation of *V*_cmax_ with further reductions of *g*_s_ to optimize *c*_i_ at these acclimation timescales. Leaf developmental adjustments of *g*_smax_ occurring at seasonal and longer timescales (step 3–4 in Fig. 3A) led to an increase in *g*_s_:*g*_smax_ returning to the long-term optimal value of γ (blue dashed line in Fig. 3A). As a result, the leaf returned to optimal combinations of photosynthetic and gas exchange traits under the new environmental conditions (step 4 in Fig. 3A, cross-section of blue dashed lines). These asynchronous leaf trait responses led to adjustments in *A*_leaf_ at each time step. After the initial increase in CO_2_, *A*_leaf_ increased by 26 % (step 0–1 in Fig. 3A, orange line in Fig. 3B). Subsequent adjustments of *g*_s_ and *V*_cmax_ from step 1 to 4 led to reductions of *A*_leaf_ of 1 and 1.6 % in steps 1–2 and 2–3, respectively (orange line in Fig. 3B).

Similar to the modelled responses to a sudden increase in CO_2_, we also explored the step-by-step responses to an increase in VPD from the initial 1 to 2 kPa (step 0–1 in Fig. 3C), while keeping all other environmental variables including temperature constant. This sudden air drying resulted in an instantaneous increase in *c*_i_:*c*_a_/χ_(optimal)_ by 9.6 % from 1.0 to 1.1 due to a decrease in χ_(optimal)_ from 0.75 to 0.69 (−8.8 %) (brown line in Fig. 3D). The subsequent reduction in stomatal aperture occurring at a timescale of minutes (step 1–2 in Fig. 3C) led to a decrease in the ratio *c*_i_:*c*_a_/χ_(optimal)_ of 2.1 % (green line in Fig. 3D). Owing to the decrease in stomatal conductance from 0.22 to 0.21 (−6.8 %) (blue line in Fig. 3D), the ratio *g*_s_:*g*_smax_ moved below the long-term optimal γ (blue dashed line in Fig. 3C). Acclimation of leaf photosynthetic capacity (step 2–3 in Fig. 3C) increased *V*_cmax_ by 3.6 % (purple line in Fig. 3D), accompanied by a decrease in *c*_i_:*c*_a_/χ_(optimal)_ of 6.8 % (green line in Fig. 3D). Our combination of models also revealed that further coordination of *g*_s_ with *V*_cmax_ following optimality principles led to an 18.8 % decrease in *g*_s_ from 0.21 to 0.17 (blue line in Fig. 3D). Finally, leaf developmental adjustments of *g*_smax_ occurring at seasonal and longer timescales (step 3–4 in Fig. 3C) led to an increase in *g*_s_:*g*_smax_ back to the long-term optimal γ (blue dashed line in Fig. 3C). As a result, the leaf returned to optimal trait combinations under the new environmental conditions (blue dashed lines cross-section in Fig. 3C). Across these four time steps, the combination of trait responses led to subsequent changes in *A*_leaf_ (orange line in Fig. 3D) that were most pronounced in relation to biochemical adjustments of *V*_cmax_ at acclimation timescales.

The time-lapse (Fig. 3D) showed the (normalized) trait responses as described previously of *c*_i_, *g*_s_ and *V*_cmax_, along with the time-lapse of *A*_leaf_. After the initial increase in VPD, *A*_leaf_ remained constant at 13.9 (orange line in Fig. 3D). From step 1 to 2 in Fig. 3C, *A*_leaf_ decreased by 0.9 % (orange line in Fig. 3D) due to the change in stomatal aperture (blue line in Fig. 3D) in response to the VPD increase. At step 2–3 in Fig. 3C, on the timescale of biochemical acclimation, *A*_leaf_ decreased further by 3 % (orange line in Fig. 3D) due to a decrease in *g*_s_ (blue line in Fig. 3D) despite an increase in *V*_cmax_ (purple line in Fig. 3D), and remained constant thereafter.

## DISCUSSION

### Literature review

We identified 17 leaf traits crucial for EEO-based modelling and determined their response mechanisms and timescales. In general, physiological and phenotypic response mechanisms were considered most relevant for modelling EEO-based trait dynamics, while evolutionary constraints limit response ranges. Leaf traits involved in physiological responses typically occur on the shortest timescale, ranging from seconds to weeks. Physiological responses primarily involve fast responses of stomatal and hydraulic movement driven by ion and water fluxes and phytohormones, which occur within existing leaf structures with relatively limited ranges. Biochemical responses require processes such as enzymatic reactions, metabolic pathways and molecule synthesis, and morphological responses require complex structural alterations (cell division, elongation, differentiation), resource allocation and genetic regulation (Smith, 2024). Logically, these adjustments require more time, hence introducing a clear separation in timescales between physiological and phenotypic adjustments in traits. However, our results suggest that coordination between physiological and phenotypic adjustments may occur at longer timescales as a consequence of optimal trait combinations in *g*_s_ and *V*_cmax_.

Mengoli *et al.* (2022) made a first attempt to separate the timescales of responses of the P-model. The authors explicitly separate the instantaneous response of photosynthesis and, on a weekly to monthly timescale, acclimated *V*_cmax_ and *J*_max_. Mengoli *et al.* (2022) also explicitly separate the fast stomatal response from acclimated χ_(optimal)_ by using a dynamic optimization of *g*_s_ operating on χ_(optimal)_. This is in line with the results of our review, where photosynthetic rate and *g*_s_ respond instantaneously, and biochemical capacities adjust on a timescale of weeks to months. However, leaf traits that respond on a developmental to evolutionary timescale pose physiological and biochemical constraints on a leaf (e.g. *g*_s_ is constrained by *g*_smax_). We argue that these structural and anatomical constraints need to also be incorporated in order to realistically model possible leaf trait combinations and the timescale at which leaf traits can respond beyond their physiological and phenotypic plasticity range.

Our framework operates at the leaf level, which therefore omits responses at whole-plant and larger scales. This excludes root traits, resource allocation strategies, soil moisture influences and nutrient availability. Also, our framework does not consider introduction of new genotypes due to migration and competition. However, this also provides an opportunity for future EEO modelling directions to extend the framework to the whole-plant level. For example, including hydraulic models in the P-model (Joshi *et al.*, 2022) can expand our proof-of-concept to whole-plant hydraulic traits. Also, research is done on extending the WLES with a root economics spectrum (Carmona *et al.*, 2021), which provides opportunities to expand our framework to the whole-plant level via assumptions on plant allometry (e.g. Shen *et al.*, 2019; Franklin *et al.*, 2020).

Our literature review reveals recurring differences in the key traits between needle-leaves and broadleaves, where needle-leaved species typically exhibit lower *A*_sat_ values and hydraulic capacities (Brodribb *et al.*, 2005, 2007), as well as lower *g*_s(operational)_ and *g*_smax_ values (Lammertsma *et al.*, 2011; Mcelwain *et al.*, 2016) than broadleaved species. Additionally, needle-leaved and broadleaved species have different vein anatomies (Brodribb *et al.*, 2005), which imposes distinct constraints on hydraulic traits and subsequent photosynthetic and gas exchange traits. Moreover, leaf habit (deciduous versus evergreen) also affects trait responses of the WLES to environmental variables, especially LMA (Wang *et al.*, 2023). This highlights the importance of considering life history of leaves over evolutionary timescales in EEO modelling.

Our illustration of leaf trait responses to changes in CO_2_ and VPD disentangle how leaves adjust their investments in leaf traits in response to a new environment to reach χ_(optimal)_ across timescales ranging from instantaneous to leaf-developmental scale and beyond. An instantaneous increase in CO_2_ mainly leads to biochemical adjustment of *c*_i_ and *V*_cmax_ to match the increased supply for assimilation (Prentice *et al.*, 2014; Quebbeman and Ramirez, 2016; Song *et al.*, 2021), while an increase in VPD leads to the most pronounced changes in *g*_s_ in order to prevent leaf desiccation while maintaining assimilation rates (Wright *et al.*, 2003; Domingues *et al.*, 2010). Surprisingly, our scenarios show a second adjustment of *g*_s_ due to biochemical feedback of *V*_cmax_ (Fig. 3A and 3C step 2–3, Fig. 3B and 3D blue line), which could be an artefact of the models used, but is surely interesting to test in controlled experiments.

Leaf functional traits show great plasticity in response to the environment, but an increase in productivity can only be achieved if certain traits and their functions remain coordinated. For example, increases in *A*_leaf_ require an increase in leaf hydraulic capacity, in order for a leaf to replace the extra loss of water through the stomata (Beerling and Franks, 2010). A potential mechanism for leaf trait coordination is cell size, which is linked to genome size in eukaryotic organisms (Faizullah *et al.*, 2021), and links to a variety of plant phenotypes, including plant structure sizes, cell metabolism and division rates, and physiological rates (Beaulieu *et al.*, 2008; Roddy *et al.*, 2020). Brodribb *et al.*, (2013)  Carins Murphy *et al.*, (2014, 2016) showed coordination between cell size and the modification of vein density and stomatal density. Gago *et al.*, (2019) found a phylogenetic signal of mesophyll conductance, potentially mediated by cell size, and Roddy *et al.* (2020) found that variation in genome size is a strong predictor of maximum photosynthetic rates across vascular plants. This is supported by Théroux-Rancourt *et al.* (2021), who showed that genome size determines the sizes and packing densities of leaf tissue cells. A large total surface area per tissue volume of mesophyll cells, which can be reached by downsizing mesophyll cells through downsizing genome size, results in higher CO_2_ diffusion rates in vascular plants (Théroux-Rancourt *et al.*, 2021). This links decreased cell size and genome downscaling (in particular for angiosperms) to increased rates of photosynthesis through coordinated changes in stomata, veins and leaf mesophyll tissue.

## CONCLUSION

The purpose of this study was to identify key leaf-level traits, their temporal constraints and their corresponding timescales of operation for improving EEO modelling. We identified 17 leaf-level traits associated with stomata, hydraulics, biochemistry, leaf morphology and leaf lifespan, which are all coordinated to operate efficiently. In particular, stomatal and hydraulic traits are tightly coupled to allow maximization of carbon assimilation rates, which may be linked by genome size. Also, strategic allocations are made in the biochemical traits *V*_cmax_ and *J*_max_, aiming to maximize photosynthesis while avoiding unnecessary and inefficient overinvestment in either trait. Leaf morphological traits are involved in the spectrum of leaf strategies, ranging from conservative to high-turnover ends. This combines traits of biochemistry and gas exchange, while also incorporating leaf lifespan and morphology into the overall strategy. A distinction becomes apparent between needle-leaves and broadleaves as well as between deciduous and evergreen leaves, and separating them in EEO-based models may be an improvement for trait response accuracy. Our conceptual framework highlighted the importance to distinguish between the separate timescales of responses in *c*_i_:*c*_a_ and χ_(optimal)_. Our approach thereby integrates the theory of plant ecophysiological responses with EEO-based modelling assuming leaves consistently strive for optimality. Optimality is reached by strategically investing in key traits, aiming to minimize the summed costs while maximizing carbon gain. Our approach suggests a way forward in connecting plant ecophysiology with vegetation modelling, thereby contributing to the improvement of EEO modelling through the separation of the timescales of trait responses.

## SUPPLEMENTARY DATA

Supplementary data are available at *Annals of Botany* online and consist of the following.

(1) ‘Literature review’ containing Table S1: Key leaf traits with their corresponding description, units, and key references as compiled from the literature review. (2) ‘Detailed modelling approach and code’. (3) ‘Tables S2–S5: Modelling output’. The full model code can also be found on GitHub: https://doi.org/10.5281/zenodo.14191170.

mcaf045_suppl_Supplementary_Data

mcaf045_suppl_Supplementary_Table_S1

mcaf045_suppl_Supplementary_Table_S2-S5
