# Correction to: Temporal constraints on leaf-level trait plasticity for next-generation land surface models

**DOI:** 10.1093/aob/mcaf224

**Published:** 2025-10-24

This is a correction to: A Odé, N G Smith, K T Rebel, H J de Boer, Temporal constraints on leaf-level trait plasticity for next-generation land surface models, *Annals of Botany*, Volume 136, Issue 2, July 2025, Pages 263–274, https://doi.org/10.1093/aob/mcaf045.

In the originally published version of this manuscript, the *y*-axis in Figure 3A and 3C was incorrectly labelled ‘*g*_s_:*g*_smax_’, instead of ‘*c*_i_:*c*_a_/χ_(optimal)_’, due to a formatting error.

The correct version of Figure 3 and its caption are presented below:

**Figure:**
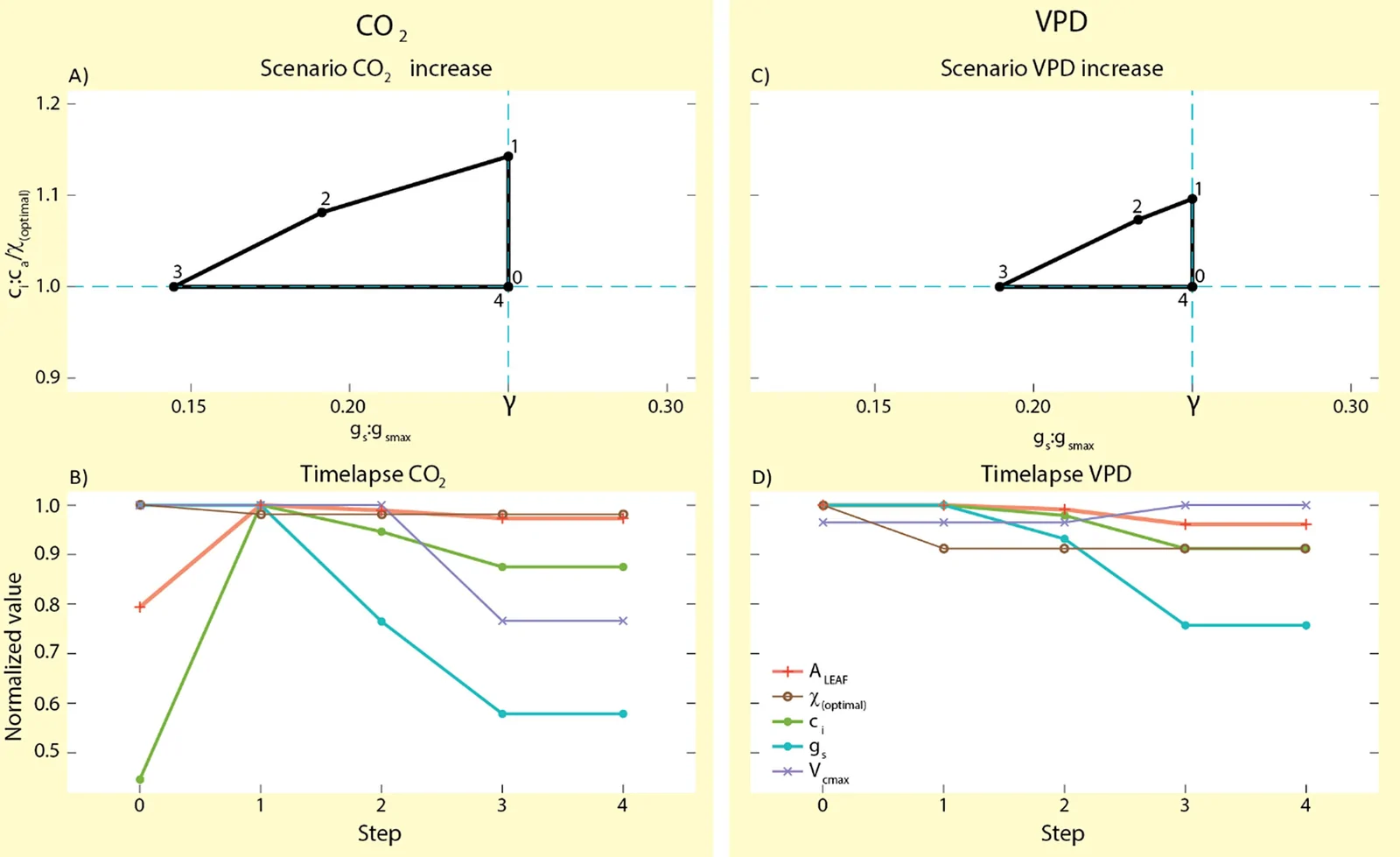


Fig. 3. Modelled scenarios to the new conceptual framework of idealized leaf gas exchange across timescales. The *x*-axis is the *g*_s_:*g*_smax_ ratio and the assumed optimal γ, and the *y*-axis describes *c*_i_:*c*_a_/χ_(optimal)_. The time-lapses illustrate individual leaf traits normalized to the maximum value on a scale of 0 to 1 over the time steps of the scenarios. Step 0 represents the initial optimized trait combination. Step 0–1 represents the near-instantaneous (seconds) response, 1–2 represents the physiological response in stomatal aperture (minutes), 2–3 represents biochemical acclimation of photosynthetic capacity (weeks) and 3–4 represents leaf developmental adjustment of stomatal anatomy (seasonal and longer). Step 4 represents the new optimized trait combination. (A) Scenario of *c*_a_ increase from 400 to 800 ppm. (B) Time-lapse of individual leaf traits *A*_leaf_, χ_(optimal)_  *c*_i_, *g*_s_ and *V*_cmax_ for the CO_2_ increase scenario. (C) Scenario of VPD increase from 1 to 2 kPa. (D) Time-lapse of individual leaf traits *A*_leaf_, χ_(optimal),_  *c*_i_, *g*_s_ and *V*_cmax_ for the VPD increase scenario.

The online version of the manuscript has been updated to include the corrected Figure 3.

