# Supplementary material for: Temporal constraints on leaf-level trait plasticity for next-generation land surface models
Source: Ann Bot. 2025 Mar 24;136(2):263–74. doi: 10.1093/aob/mcaf045 (PMC12445853; doi:10.1093/aob/mcaf045)
Supplement: mcaf045_suppl_Supplementary_Data [file mcaf045_suppl_supplementary_data.docx]

**[Supplementary Information for:](https://www.nature.com/nature/for-authors/supp-info)**

**Temporal constraints on leaf-level trait plasticity for next-generation land surface models**

A Odé, NG Smith, KT Rebel, HJ de Boer

**Framework scenarios: modelling approach**

Leaf traits responses to CO_2_ and VPD were illustrated by framework scenarios as a proof-of-concept. We assumed that the starting point (step 0) and end point (step 4) corresponds to the optimal leaf trait combination for the prevailing and new environment, respectively. To calculate the start and end points, we have calculated χ_(optimal)__start_,_ χ_(optimal)__end, V_cmax__start, and V_cmax__end from the P-model using the “optimal_vcmax_R” repository as reported in Smith et al. (2019); DOI 10.5281/zenodo.14186765. Environmental input variables were: temperature 25 degrees Celsius, PPFD 800 µmol·m-2·s-1, VPD 1 kPa, Ca 400 ppm. For the CO_2_ scenario we assigned a Ca_start of 400 and Ca_end of 800, and for the VPD scenarios VPD_start of 1 and VPD_end of 2. Between the start point and the end point of the leaf, leaf trait responses occur on physiological response time scales, acclimation time scales, and evolutionary time scales. We simulated these intermediate steps using the R ‘plantecophys’ package (version 1.4-6), with the Photosyn function (Duursma 2015) as follows:

- Step 0: we calculated c_i__start from χ_(optimal)__start and C_a_ as:

$C_{i}\_start = \chi_{(optimal)}\_\_start * C_{a}\_start$

Then we calculated the start leaf trait values using the Photosyn function with input C_i__start, V_cmax__start and the initial environmental conditions.

- Step 0-1: represents the instantaneous responses of leaf traits to a change in the environment, so the change in C_i_. We calculated the new C_i_ for the changed environmental conditions, with the input g_s__start calculated at the previous step, and V_cmax__start, since g_s_ and V_cmax_ have not responded yet on this physiological response time scale. From this step onwards, the value χ_(optimal)__end applies for the new environment.
- Step 1-2: represents the change in stomatal aperture on a time scale of minutes. We calculated g_s_ with input V_cmax__start, since V_cmax_ has not yet responded on this time scale of the physiological stomatal response, and the environmental variables of the new environment.
- Step 2-3: represents the time scale of biochemical acclimation. We calculated a new C_i_, C_i__end, with χ_(optimal)__end and C_a__end similar as in step 0. Then we simulate this step with input C_i__end, V_cmax__end, and the environmental variables of the changed environment.
- Step 3-4: represents trait changes on a developmental time scale. In line with the assumption that leaves aim to conserve γ, we calculated the g_smax_ at the start as g_s_/0.25. This start g_smax__start remains constant until step 4. Then in step 4, we calculate the new adjusted g_smax__end as the end point g_s_ value/0.25.

Simulations were plotted by calculating the x-axis coordinates as g_s_/g_smax_ for every step, and coordinates of the y-axis as c_i_:c_a_/ χ_(optimal)_ for every step.

**Full code as plain text**

The full code can also be found on Github: https://doi.org/10.5281/zenodo.14191170

# Code for modelling idealized leaf trait responses to environmental changes as used in:

# Temporal constraints on leaf-level trait plasticity for next-generation land surface models

# Odé at al. (yr), in preparation

#### variable key ####

# P-model variables:

#tg_c: acclimated temperature (degrees Celsius)

#vpdo: vapor pressure deficit at sea level (kPa)

#cao: atmospheric CO2 at sea level (umol mol-1)

#paro: photosynthetically active radiation at sea level (µmol m-2 s-1)

#gs: stomatal conductance to water vapour

#Ci: intercellular CO2 concentration (ppm)

#Chi: χ(optimal) (unitless)

#Vcmax: maximum carboxylation rate (µmol·m-2·s-1)

#ALEAF: net assimilation rate (µmol·m-2·s-1)

# Photosyn variables:

#Ci: intercellular CO2 concentration (ppm)

#VPD: vapour pressure deficit (kPa)

#Tleaf: leaf temperature (degrees Celsius)

#Ca: atmospheric CO2 concentration (ppm)

#PPFD: photosynthetic photon flux density (µmol m-2·s-1)

#GS: stomatal conductance to water vapour

# Load libraries

library(R.utils)

library(plantecophys)

library(ggplot2)

library(tidyverse)

# Load necessary functions using path to own working directory

sourceDirectory("functions", modifiedOnly = FALSE)

source("calc_optimal_vcmax.R")

#### CO2 scenario ####

#### Part 1: framework scenario ####

# Choose environmental start- and end variables #

cao_start<-400

cao_end<- 800

vpdo_start <- 1

vpdo_end <- 1

paro_start <- 800

paro_end <- 800

t_start<-25

t_end<-25

# Calculate start (optimal) trait values#

rpmodel_step0<-data.frame()

rpmodel_step0<-calc_optimal_vcmax(cao=cao_start, par=paro_start, vpdo=vpdo_start, tg_c=t_start) #calculate start values

vcmax_start<-rpmodel_step0$vcmax[1] #Store start Vcmax value

chi_start<-rpmodel_step0$chi[1] #Store start chi value

# Calculate end (optimal) trait values #

step_end_rpmodel <-calc_optimal_vcmax(cao=cao_end, par=paro_end, vpdo=vpdo_end, tg_c=t_end) #calculate end values

vcmax_end<-step_end_rpmodel$vcmax[1] #Store end Vcmax value

chi_end<-step_end_rpmodel$chi[1] #Store end chi value

# Step 0: Start conditions

step0 <- Photosyn(Ca=cao_start, PPFD=paro_start, Tleaf=t_start, VPD=vpdo_start, Vcmax=vcmax_start)

ci_start<-chi_start*step0$Ca

step0 <- Photosyn(Ci=ci_start, Ca=cao_start, PPFD=paro_start, Tleaf=t_start, VPD=vpdo_start, Vcmax=vcmax_start, gsmodel="BBOpti")

gs_start<-step0$GS

# Step 0-1: Instantaneous response

step1 <- Photosyn(GS=gs_start, Ca=cao_end, Vcmax=vcmax_start, VPD=vpdo_end, PPFD=paro_end, Tleaf=t_end, gsmodel="BBOpti")

# Step 1-2: Stomatal aperture response

step2 <- Photosyn(cao_end, Vcmax=vcmax_start, VPD=vpdo_end, PPFD=paro_end, Tleaf=t_end, gsmodel="BBOpti")

# Step 2-3: Biochemical acclimation response

step3 <- Photosyn(Ca=cao_end, VPD=vpdo_end, PPFD=paro_end, Vcmax=vcmax_end, Tleaf=t_end, gsmodel="BBOpti")

ci_end<-chi_end*step3$Ca

step3 <- Photosyn(Ci=ci_end, Ca=cao_end, VPD=vpdo_end, PPFD=paro_end, Vcmax=vcmax_end, Tleaf=t_end, gsmodel="BBOpti")

# Step 4: Developmental response (same as step 3, but we will adjust gsmax in line 89)

step4 <- step3

# Combine all steps in one dataframe

combined <- rbind(step0, step1, step2, step3, step4)

# Add Chi and Vcmax columns to dataframe

combined$Chi <- c(chi_start, rep(chi_end, 4))

combined$Vcmax <- c(vcmax_start, vcmax_start, vcmax_start, vcmax_end, vcmax_end)

# Add gsmax values to dataframe

gsmax_start <- step0$GS / 0.25

gsmax_end <- step3$GS / 0.25

combined$gsmax <- c(gsmax_start, gsmax_start, gsmax_start, gsmax_start, gsmax_end)

# Calculate x- and y-axes

combined$xaxis <- combined$GS / combined$gsmax

combined$yaxis <- (combined$Ci / c(cao_start, rep(cao_end, 4))) / combined$Chi

# Label columns as steps and reorder

combined$Step <- c("0", "1", "2", "3", "4")

combined <- combined[, c("Step", "Chi","Ci","GS","Vcmax","gsmax", "xaxis", "yaxis", names(combined)[!names(combined) %in% c("Step", "GS", "Ci", "Chi", "Vcmax", "gsmax", "xaxis", "yaxis")])]

# Plot the scenario #

ggplot(combined, aes(x=xaxis, y=yaxis, label=Step)) +

geom_point() +

geom_path(linetype='solid', linewidth=1.5) +

theme_classic(base_size = 20) +

xlab("gs/gsmax") + ylab("ci:ca/chi") +

ggtitle("Scenario title") +

geom_hline(yintercept=1, color='Blue', linewidth=1, linetype='dashed') +

geom_vline(xintercept=0.25, color='Blue', linewidth=1, linetype='dashed') +

ylim(0.90,1.2) + xlim(0.12,0.3) +

geom_text(hjust=-0.2, vjust=-0.5, show.legend = FALSE) # Add this line to show labels

#### Part 2: timelapse of normalized leaf trait values ####

# Normalize trait values to the maximum value per trait #

combined <- combined %>%

mutate(GS_normalized = GS / max(GS, na.rm = TRUE))

combined <- combined %>%

mutate(ALEAF_normalized = ALEAF / max(ALEAF, na.rm = TRUE))

combined <- combined %>%

mutate(Ci_normalized = Ci / max(Ci, na.rm = TRUE))

combined <- combined %>%

mutate(Vcmax_normalized = Vcmax / max(Vcmax, na.rm = TRUE))

combined <- combined %>%

mutate(chi_normalized = Chi / max(Chi, na.rm = TRUE))

# Create the data frame with normalized values and pivot to correct format #

timelapse_dataframe <- combined %>%

select(Step, GS_normalized, ALEAF_normalized, Ci_normalized, Vcmax_normalized, chi_normalized) %>%

pivot_longer(cols = c(GS_normalized, ALEAF_normalized, Ci_normalized, Vcmax_normalized, chi_normalized),

names_to = "Variable",

values_to = "Normalized_value") %>%

mutate(Variable = sub("_normalized", "", Variable))

# Create a plot for the timelapse grouping the values per trait value #

timelapse_dataframe<-ggplot(timelapse_dataframe, aes(x=Step, y=Normalized_value, color=Variable)) +

geom_line(linewidth = 1, aes(group=Variable)) +

geom_point(size=3)+

theme_bw()+

ggtitle("Timelapse CO2 scenario")+

theme_classic(base_size = 15)

print(timelapse_dataframe)

#### VPD scenario ####

#### Part 1: framework scenario ####

# Choose environmental start- and end variables #

cao_start<-400

cao_end<- 400

vpdo_start <- 1

vpdo_end <- 2

paro_start <- 800

paro_end <- 800

t_start<-25

t_end<-25

# Calculate start (optimal) trait values#

rpmodel_step0<-data.frame()

rpmodel_step0<-calc_optimal_vcmax(cao=cao_start, par=paro_start, vpdo=vpdo_start, tg_c=t_start) #calculate start values

vcmax_start<-rpmodel_step0$vcmax[1] #Store start Vcmax value

chi_start<-rpmodel_step0$chi[1] #Store start chi value

# Calculate end (optimal) trait values #

step_end_rpmodel <-calc_optimal_vcmax(cao=cao_end, par=paro_end, vpdo=vpdo_end, tg_c=t_end) #calculate end values

vcmax_end<-step_end_rpmodel$vcmax[1] #Store end Vcmax value

chi_end<-step_end_rpmodel$chi[1] #Store end chi value

# Step 0: Start conditions

step0 <- Photosyn(Ca=cao_start, PPFD=paro_start, Tleaf=t_start, VPD=vpdo_start, Vcmax=vcmax_start)

ci_start<-chi_start*step0$Ca

step0 <- Photosyn(Ci=ci_start, Ca=cao_start, PPFD=paro_start, Tleaf=t_start, VPD=vpdo_start, Vcmax=vcmax_start, gsmodel="BBOpti")

gs_start<-step0$GS

# Step 0-1: Instantaneous response

step1 <- Photosyn(GS=gs_start, Ca=cao_end, Vcmax=vcmax_start, VPD=vpdo_end, PPFD=paro_end, Tleaf=t_end, gsmodel="BBOpti")

# Step 1-2: Stomatal aperture response

step2 <- Photosyn(cao_end, Vcmax=vcmax_start, VPD=vpdo_end, PPFD=paro_end, Tleaf=t_end, gsmodel="BBOpti")

# Step 2-3: Biochemical acclimation response

step3 <- Photosyn(Ca=cao_end, VPD=vpdo_end, PPFD=paro_end, Vcmax=vcmax_end, Tleaf=t_end, gsmodel="BBOpti")

ci_end<-chi_end*step3$Ca

step3 <- Photosyn(Ci=ci_end, Ca=cao_end, VPD=vpdo_end, PPFD=paro_end, Vcmax=vcmax_end, Tleaf=t_end, gsmodel="BBOpti")

# Step 4: Developmental response (same as step 3, but we will adjust gsmax in line 89)

step4 <- step3

# Combine all steps in one dataframe

combined <- rbind(step0, step1, step2, step3, step4)

# Add Chi and Vcmax columns to dataframe

combined$Chi <- c(chi_start, rep(chi_end, 4))

combined$Vcmax <- c(vcmax_start, vcmax_start, vcmax_start, vcmax_end, vcmax_end)

# Add gsmax values to dataframe

gsmax_start <- step0$GS / 0.25

gsmax_end <- step3$GS / 0.25

combined$gsmax <- c(gsmax_start, gsmax_start, gsmax_start, gsmax_start, gsmax_end)

# Calculate x- and y-axes

combined$xaxis <- combined$GS / combined$gsmax

combined$yaxis <- (combined$Ci / c(cao_start, rep(cao_end, 4))) / combined$Chi

# Label columns as steps and reorder

combined$Step <- c("0", "1", "2", "3", "4")

combined <- combined[, c("Step", "Chi","Ci","GS","Vcmax","gsmax", "xaxis", "yaxis", names(combined)[!names(combined) %in% c("Step", "GS", "Ci", "Chi", "Vcmax", "gsmax", "xaxis", "yaxis")])]

# Plot the scenario #

ggplot(combined, aes(x=xaxis, y=yaxis, label=Step)) +

geom_point() +

geom_path(linetype='solid', linewidth=1.5) +

theme_classic(base_size = 20) +

xlab("gs/gsmax") + ylab("ci:ca/chi") +

ggtitle("Scenario title") +

geom_hline(yintercept=1, color='Blue', linewidth=1, linetype='dashed') +

geom_vline(xintercept=0.25, color='Blue', linewidth=1, linetype='dashed') +

ylim(0.90,1.2) + xlim(0.12,0.3) +

geom_text(hjust=-0.2, vjust=-0.5, show.legend = FALSE) # Add this line to show labels

#### Part 2: timelapse of normalized leaf trait values ####

# Normalize trait values to the maximum value per trait #

combined <- combined %>%

mutate(GS_normalized = GS / max(GS, na.rm = TRUE))

combined <- combined %>%

mutate(ALEAF_normalized = ALEAF / max(ALEAF, na.rm = TRUE))

combined <- combined %>%

mutate(Ci_normalized = Ci / max(Ci, na.rm = TRUE))

combined <- combined %>%

mutate(Vcmax_normalized = Vcmax / max(Vcmax, na.rm = TRUE))

combined <- combined %>%

mutate(chi_normalized = Chi / max(Chi, na.rm = TRUE))

# Create the data frame with normalized values and pivot to correct format #

timelapse_dataframe <- combined %>%

select(Step, GS_normalized, ALEAF_normalized, Ci_normalized, Vcmax_normalized, chi_normalized) %>%

pivot_longer(cols = c(GS_normalized, ALEAF_normalized, Ci_normalized, Vcmax_normalized, chi_normalized),

names_to = "Variable",

values_to = "Normalized_value") %>%

mutate(Variable = sub("_normalized", "", Variable))

# Create a plot for the timelapse grouping the values per trait value #

timelapse_dataframe<-ggplot(timelapse_dataframe, aes(x=Step, y=Normalized_value, color=Variable)) +

geom_line(linewidth = 1, aes(group=Variable)) +

geom_point(size=3)+

theme_bw()+

ggtitle("Timelapse VPD")+

theme_classic(base_size = 15)

print(timelapse_dataframe)

**Literature cited:**

**Duursma RA**. **2015**. Plantecophys - An R package for analysing and modelling leaf gas exchange data. *PLoS ONE* **10**.

**Smith NG, Keenan TF, Colin Prentice I, *et al.*** **2019**. Global photosynthetic capacity is optimized to the environment. *Ecology Letters* **22**: 506–517.
