# Supplementary material for: Temporal constraints on leaf-level trait plasticity for next-generation land surface models
Source: Ann Bot. 2025 Mar 24;136(2):263–74. doi: 10.1093/aob/mcaf045 (PMC12445853; doi:10.1093/aob/mcaf045)
Supplement: mcaf045_suppl_Supplementary_Table_S1 [file mcaf045_suppl_supplementary_table_s1.docx]

**[Supplementary Information for:](https://www.nature.com/nature/for-authors/supp-info)**

**Temporal constraints on leaf-level trait plasticity for next-generation land surface models**

A Odé, NG Smith, KT Rebel, HJ de Boer

**Table S1:** Overview of key leaf-level traits involved in gas exchange and photosynthesis with their corresponding units, definition, and key references, compiled from the literature review. Traits with an asterisk are incorporated in the P-model.

| Leaf trait | Units | Definition | Key literature |
| --- | --- | --- | --- |
| g_s*_ | mol·m^-2^·s^-1^ | Stomatal conductance | (Vico *et al.* 2011; Franks *et al.* 2012; Mcelwain *et al.* 2016; Elliott-Kingston *et al.* 2016; Murray *et al.* 2020) |
| c_i_* | Pa or ppm | Intercellular CO_2_ concentration | (Farquhar *et al.* 1980; Farquhar and Sharkey 1982; Franks and Farquhar 2007) |
| g_s(operational)_ | mol·m^-2^·s^-1^ | Operational stomatal conductance | (Franks *et al.* 2012; Mcelwain *et al.* 2016; Murray *et al.* 2020) |
| χ_(optimal)_* | unitless | Optimal c_i_:c_a_ ratio as calculated from the P-model | (Prentice *et al.* 2014; Stocker *et al.* 2020; Harrison *et al.* 2021) |
| V_cmax_* | mol·m^-2^·s^-1^ | Maximum carboxylation rate | (Smith *et al.* 2019; Smith and Keenan 2020) |
| J_max_* | mol·m^-2^·s^-1^ | Maximum electron transport rate | (Smith *et al.* 2019; Smith and Keenan 2020) |
| g_smax_ | mol·m^-2^·s^-1^ | Maximum anatomical stomatal conductance. g_smax_ is determined by stomatal pore length, stomatal pore depth, and stomatal density | (Franks and Beerling 2009; De Boer *et al.* 2011; Lammertsma *et al.* 2011; Drake *et al.* 2013; de Boer, Price, *et al.* 2016) |
| Stomatal density | stomata·mm^-2^ | Number of stomata per leaf area | See g_smax_ |
| Stomatal size | µm | Stomatal length multiplied by stomatal width | See g_smax_ |
| VLA | mm·mm^−2^ | Vein length per unit leaf area (vein density) | (Sack and Holbrook 2006; Carins Murphy *et al.* 2012; de Boer, Drake, *et al.* 2016) |
| LVA | mm^3^·mm^-2^ | Leaf thickness; leaf volume per area / leaf volume to area ratio | (Wright *et al.* 2004; Poorter *et al.* 2009) |
| LD | g·cm^-3^ | Leaf dry mass per leaf volume | (Wright *et al.* 2004; Poorter *et al.* 2009) |
| LL | Months | Leaf lifespan | (Vincent 2006; Wang *et al.* 2023) |
| Ψ_leaf_ | MPa or kPa | Leaf water potential | (Sack and Holbrook 2006; Salvi *et al.* 2022) |
| Leaf hydraulic capacity | mmol·m^−2^ ·s^−1^·MPa^−1^ | Maximum leaf hydraulic conductance for hydrated leaf | (Sack and Holbrook 2006; Salvi *et al.* 2022) |
| K_leaf_ | mmol m^−2^ ·s^−1^·MPa^−1^ | Leaf hydraulic conductance; ratio of water flow rate to the water potential difference across the leaf | (Brodribb *et al.* 2005, 2007; Sack and Holbrook 2006; Scoffoni *et al.* 2016) |
| Photosynthetic pathway* | categorical | C_3_, C_4_, or CAM | (Sage 2004; Yamori *et al.* 2014; Sage *et al.* 2023) |
| Genome size | Gbp | Amount of DNA present in diploid (2C) cell nucleus set (Gigabase pairs) | (Beaulieu *et al.* 2008; Roddy *et al.* 2020; Théroux-Rancourt *et al.* 2021; Faizullah *et al.* 2021) |

**Literature cited**

**Beaulieu JM, Leitch IJ, Patel S, Pendharkar A, Knight CA**. **2008**. Genome size is a strong predictor of cell size and stomatal density in angiosperms. *New Phytologist* **179**: 975–986.

**de Boer HJ, Drake PL, Wendt E, *et al.*** **2016**. Apparent overinvestment in leaf venation relaxes leaf morphological constraints on photosynthesis in arid habitats. *Plant Physiology* **172**: 2286–2299.

**De Boer HJ, Lammertsma EI, Wagner-Cremer F, Dilcher DL, Wassen MJ, Dekker SC**. **2011**. Climate forcing due to optimization of maximal leaf conductance in subtropical vegetation under rising CO2. *Proceedings of the National Academy of Sciences of the United States of America* **108**: 4041–4046.

**de Boer HJ, Price CA, Wagner-Cremer F, Dekker SC, Franks PJ, Veneklaas EJ**. **2016**. Optimal allocation of leaf epidermal area for gas exchange. *New Phytologist* **210**: 1219–1228.

**Brodribb TJ, Feild TS, Jordan GJ**. **2007**. Leaf maximum photosynthetic rate and venation are linked by hydraulics. *Plant Physiology* **144**: 1890–1898.

**Brodribb TJ, Holbrook NM, Zwieniecki MA, Palma B**. **2005**. Leaf hydraulic capacity in ferns, conifers and angiosperms: Impacts on photosynthetic maxima. *New Phytologist* **165**: 839–846.

**Carins Murphy MR, Jordan GJ, Brodribb TJ**. **2012**. Differential leaf expansion can enable hydraulic acclimation to sun and shade. *Plant, Cell and Environment* **35**: 1407–1418.

**Drake PL, Froend RH, Franks PJ**. **2013**. Smaller, faster stomata: Scaling of stomatal size, rate of response, and stomatal conductance. *Journal of Experimental Botany* **64**: 495–505.

**Elliott-Kingston C, Haworth M, Yearsley JM, Batke SP, Lawson T, McElwain JC**. **2016**. Does size matter? Atmospheric CO2 may be a stronger driver of stomatal closing rate than stomatal size in taxa that diversified under low CO2. *Frontiers in Plant Science* **7**.

**Faizullah L, Morton JA, Hersch-Green EI, Walczyk AM, Leitch AR, Leitch IJ**. **2021**. Exploring environmental selection on genome size in angiosperms. *Trends in Plant Science* **26**: 1039–1049.

**Farquhar GD, von Caemmerer S, Berry JA**. **1980**. A biochemical model of photosynthetic CO2 assimilation in leaves of C3 species. *Planta* **149**: 78–90.

**Farquhar G D, Sharkey T D**. **1982**. Stomatal Conductance and Photosynthesis. *Annual Review of Plant Physiology* **33**: 317–345.

**Franks PJ, Beerling DJ**. **2009**. Maximum leaf conductance driven by CO2 effects on stomatal size and density over geologic time. *Proceedings of the National Academy of Sciences of the United States of America* **106**: 10343–10347.

**Franks PJ, Farquhar GD**. **2007**. The mechanical diversity of stomata and its significance in gas-exchange control. *Plant Physiology* **143**: 78–87.

**Franks PJ, Leitch IJ, Ruszala EM, Hetherington AM, Beerling DJ**. **2012**. Physiological framework for adaptation of stomata to CO2 from glacial to future concentrations. *Philosophical Transactions of the Royal Society B: Biological Sciences* **367**: 537–546.

**Harrison SP, Cramer W, Franklin O, *et al.*** **2021**. Eco-evolutionary optimality as a means to improve vegetation and land-surface models. *New Phytologist* **231**: 2125–2141.

**Lammertsma EI, De Boer HJ, Dekker SC, Dilcher DL, Lotter AF, Wagner-Cremer F**. **2011**. Global CO2 rise leads to reduced maximum stomatal conductance in Florida vegetation. *Proceedings of the National Academy of Sciences of the United States of America* **108**: 4035–4040.

**Mcelwain JC, Yiotis C, Lawson T**. **2016**. Using modern plant trait relationships between observed and theoretical maximum stomatal conductance and vein density to examine patterns of plant macroevolution. *New Phytologist* **209**: 94–103.

**Murray M, Soh WK, Yiotis C, Spicer RA, Lawson T, McElwain JC**. **2020**. Consistent relationship between field-measured stomatal conductance and theoretical maximum stomatal conductance in C3 woody angiosperms in four major biomes. *International Journal of Plant Sciences* **181**: 142–154.

**Poorter H, Niinemets Ü, Poorter L, Wright IJ, Villar R**. **2009**. Causes and consequences of variation in leaf mass per area (LMA): A meta-analysis. *New Phytologist* **182**: 565–588.

**Prentice IC, Dong N, Gleason SM, Maire V, Wright IJ**. **2014**. Balancing the costs of carbon gain and water transport: Testing a new theoretical framework for plant functional ecology. *Ecology Letters* **17**: 82–91.

**Roddy AB, Théroux-Rancourt G, Abbo T, *et al.*** **2020**. The scaling of genome size and cell size limits maximum rates of photosynthesis with implications for ecological strategies. *International Journal of Plant Sciences* **181**: 75–87.

**Sack L, Holbrook NM**. **2006**. Leaf hydraulics. *Annual Review of Plant Biology* **57**: 361–381.

**Sage RF**. **2004**. The evolution of C 4 photosynthesis. *New Phytologist* **161**: 341–370.

**Sage RF, Gilman IS, Smith JAC, Silvera K, Edwards EJ**. **2023**. Atmospheric CO2 decline and the timing of CAM plant evolution. *Annals of Botany* **132**: 753–770.

**Salvi AM, Gosetti SG, Smith DD, Adams MA, Givnish TJ, McCulloh KA**. **2022**. Hydroscapes, hydroscape plasticity and relationships to functional traits and mesophyll photosynthetic sensitivity to leaf water potential in Eucalyptus species. *Plant Cell and Environment* **45**: 2573–2588.

**Scoffoni C, Chatelet DS, Pasquet-Kok J, *et al.*** **2016**. Hydraulic basis for the evolution of photosynthetic productivity. *Nature Plants* **2**.

**Smith NG, Keenan TF**. **2020**. Mechanisms underlying leaf photosynthetic acclimation to warming and elevated CO2 as inferred from least-cost optimality theory. *Global Change Biology* **26**: 5202–5216.

**Smith NG, Keenan TF, Colin Prentice I, *et al.*** **2019**. Global photosynthetic capacity is optimized to the environment. *Ecology Letters* **22**: 506–517.

**Stocker BD, Wang H, Smith NG, *et al.*** **2020**. P-model v1.0: An optimality-based light use efficiency model for simulating ecosystem gross primary production. *Geoscientific Model Development* **13**: 1545–1581.

**Théroux-Rancourt G, Roddy AB, Earles JM, *et al.*** **2021**. Maximum CO 2 diffusion inside leaves is limited by the scaling of cell size and genome size. *Proceedings of the Royal Society B: Biological Sciences* **288**.

**Vico G, Manzoni S, Palmroth S, Katul G**. **2011**. Effects of stomatal delays on the economics of leaf gas exchange under intermittent light regimes. *New Phytologist* **192**: 640–652.

**Vincent G**. **2006**. Leaf life span plasticity in tropical seedlings grown under contrasting light regimes. *Annals of Botany* **97**: 245–255.

**Wang H, Prentice IC, Wright IJ, *et al.*** **2023**. Leaf economics fundamentals explained by optimality principles. *Science Advances* **9**.

**Wright IJ, Reich PB, Westoby M, *et al.*** **2004**. The worldwide leaf economics spectrum. *Nature* **428**: 821–827.

**Yamori W, Hikosaka K, Way DA**. **2014**. Temperature response of photosynthesis in C3, C4, and CAM plants: Temperature acclimation and temperature adaptation. *Photosynthesis Research* **119**: 101–117.
