# Supplementary material for: Temporal constraints on leaf-level trait plasticity for next-generation land surface models
Source: Ann Bot. 2025 Mar 24;136(2):263–74. doi: 10.1093/aob/mcaf045 (PMC12445853; doi:10.1093/aob/mcaf045)
Supplement: mcaf045_suppl_Supplementary_Table_S2-S5 [file mcaf045_suppl_supplementary_table_s2-s5.docx]

**[Supplementary Information for:](https://www.nature.com/nature/for-authors/supp-info)**

**Temporal constraints on leaf-level trait plasticity for next-generation land surface models**

A Odé, NG Smith, KT Rebel, HJ de Boer

**Model output**

Variables:

GS = stomatal conductance to water vapour (mol·m^-2^·s^-1^), Ci = intercellular CO_2_ concentration (ppm), Chi = χ_(optimal)_ (unitless), Vcmax = maximum carboxylation rate (µmol·m^-2^·s^-1^), gsmax = maximum anatomical stomatal conductance (mol·m^-2^·s^-1^), ALEAF = net assimilation rate (µmol·m^-2^·s^-1^), VPD = vapour pressure deficit (kPa), Tleaf = leaf temperature (degrees Celsius), Ca = atmospheric CO_2_ concentration (ppm), PPFD = photosynthetic photon flux density (µmol·m^-2^·s^-1^), Patm = atmospheric pressure (kPa).

| Step | Chi | Ci | GS | Vcmax | gsmax | ALEAF | VPD | Tleaf | Ca | PPFD | Patm | xaxis | yaxis |
| --- | --- | --- | --- | --- | --- | --- | --- | --- | --- | --- | --- | --- | --- |
| 0 | 0.754 | 301.787 | 0.222 | 99.825 | 0.888 | 13.894 | 1 | 25 | 400 | 800 | 100 | 0.250 | 1.000 |
| 1 | 0.740 | 676.293 | 0.222 | 99.825 | 0.888 | 17.501 | 1 | 25 | 800 | 800 | 100 | 0.250 | 1.143 |
| 2 | 0.740 | 640.000 | 0.170 | 99.825 | 0.888 | 17.315 | 1 | 25 | 800 | 800 | 100 | 0.191 | 1.081 |
| 3 | 0.740 | 591.821 | 0.128 | 76.482 | 0.888 | 17.035 | 1 | 25 | 800 | 800 | 100 | 0.145 | 1.000 |
| 4 | 0.740 | 591.821 | 0.128 | 76.482 | 0.514 | 17.035 | 1 | 25 | 800 | 800 | 100 | 0.250 | 1.000 |

**Table S2:** scenario output from simulating CO_2_ increase of 400 ppm to 800 ppm, corresponding to Fig.3A in the main paper. Grey shading is used to highlight the steps at which a leaf trait changes from onwards.

| Step |  | GS_normalized | ALEAF_normalized | Ci_normalized | Vcmax_normalized | Chi_normalized |
| --- | --- | --- | --- | --- | --- | --- |
| 0 |  | 1.000 | 0.794 | 0.446 | 1.000 | 1.000 |
| 1 |  | 1.000 | 1.000 | 1.000 | 1.000 | 0.981 |
| 2 |  | 0.765 | 0.989 | 0.946 | 1.000 | 0.981 |
| 3 |  | 0.578 | 0.973 | 0.875 | 0.766 | 0.981 |
| 4 |  | 0.578 | 0.973 | 0.875 | 0.766 | 0.981 |

**Table S3:** timelapse output from simulating CO_2_ increase of 400 ppm to 800 ppm, corresponding to Fig.3B in the main paper

| Step | Chi | Ci | GS | Vcmax | gsmax | ALEAF | VPD | Tleaf | Ca | PPFD | Patm | xaxis | yaxis |
| --- | --- | --- | --- | --- | --- | --- | --- | --- | --- | --- | --- | --- | --- |
| 0 | 0.754 | 301.787 | 0.222 | 99.825 | 0.888 | 13.894 | 1 | 25 | 400 | 800 | 100 | 0.250 | 1.000 |
| 1 | 0.688 | 301.797 | 0.222 | 99.825 | 0.888 | 13.893 | 2 | 25 | 400 | 800 | 100 | 0.250 | 1.096 |
| 2 | 0.688 | 295.518 | 0.207 | 99.825 | 0.888 | 13.774 | 2 | 25 | 400 | 800 | 100 | 0.233 | 1.073 |
| 3 | 0.688 | 275.305 | 0.168 | 103.414 | 0.888 | 13.356 | 2 | 25 | 400 | 800 | 100 | 0.189 | 1.000 |
| 4 | 0.688 | 275.305 | 0.168 | 103.414 | 0.673 | 13.356 | 2 | 25 | 400 | 800 | 100 | 0.250 | 1.000 |

**Table S4:** scenario output from simulating VPD increase form 1 kPa to 2 kPa, corresponding to Fig.3C in the main paper. Grey shading is used to highlight the steps at which a leaf trait changes from onwards.

| Step |  | GS_normalized | ALEAF_normalized | Ci_normalized | Vcmax_normalized | Chi_normalized |
| --- | --- | --- | --- | --- | --- | --- |
| 0 |  | 1.000 | 1.000 | 1.000 | 0.965 | 1.000 |
| 1 |  | 1.000 | 1.000 | 1.000 | 0.965 | 0.912 |
| 2 |  | 0.932 | 0.991 | 0.979 | 0.965 | 0.912 |
| 3 |  | 0.757 | 0.961 | 0.912 | 1.000 | 0.912 |
| 4 |  | 0.757 | 0.961 | 0.912 | 1.000 | 0.912 |

**Table S5:** timelapse output from simulating VPD increase form 1 to 2 kPa, corresponding to Fig.3D in the main paper
